# Omicron mutations increase interdomain interactions and reduce epitope exposure in the SARS-CoV-2 spike

**DOI:** 10.1016/j.isci.2023.105981

**Published:** 2023-01-20

**Authors:** Miłosz Wieczór, Phu K. Tang, Modesto Orozco, Pilar Cossio

**Affiliations:** 1 Institute for Research in Biomedicine (IRB Barcelona), Barcelona Institute of Science and Technology, 08028 Barcelona, Spain; 2 Department of Physical Chemistry, Gdansk University of Technology, 80-233 Gdansk, Poland; 3 Center for Computational Mathematics, Flatiron Institute, New York, NY 10010, USA; 4 Center for Computational Biology, Flatiron Institute, New York, NY 10010, USA; 5 Department of Biochemistry and Biomedicine, University of Barcelona, 08007 Barcelona, Spain

**Keywords:** Computational molecular modeling, Microbiology, Structural biology, Bioinformatics

## Abstract

Omicron BA.1 is a highly infectious variant of SARS-CoV-2 that carries more than thirty mutations on the spike protein in comparison to the Wuhan wild type (WT). Some of the Omicron mutations, located on the receptor-binding domain (RBD), are exposed to the surrounding solvent and are known to help evade immunity. However, the impact of buried mutations on the RBD conformations and on the mechanics of the spike opening is less evident. Here, we use all-atom molecular dynamics (MD) simulations with metadynamics to characterize the thermodynamic RBD-opening ensemble, identifying significant differences between WT and Omicron. Specifically, the Omicron mutations S371L, S373P, and S375F make more RBD interdomain contacts during the spike’s opening. Moreover, Omicron takes longer to reach the transition state than WT. It stabilizes up-state conformations with fewer RBD epitopes exposed to the solvent, potentially favoring immune or antibody evasion.

## Highlights

• The RBD in Omicron spike samples fewer conformations than in original SARS-CoV-2
• 3 clustered hydrophobic mutations stabilize the handrail opening motion of RBD
• The tighter interaction between RBDs slows down the opening kinetics in Omicron
• The altered conformational ensemble of the RBD reduces epitope accessibility

## Graphical abstract

**Figure:**
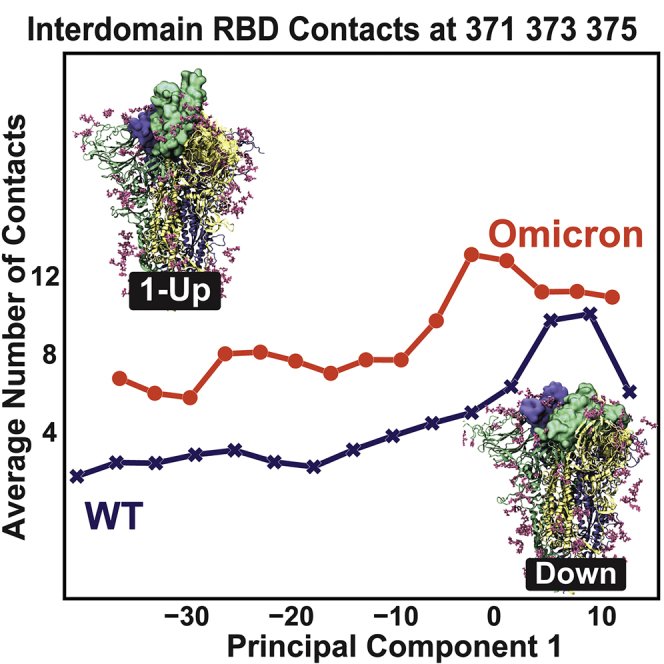

## Introduction

At the end of 2019, SARS-CoV-2 emerged in a local market in Wuhan, China and quickly spread worldwide, so that in March 2020, the WHO declared COVID-19 a global pandemic.^1^^,^^2^^,^^3^ Since then, the pandemic has claimed more than six million casualties.^2^ Thanks to global efforts, scientists quickly identified the spike protein on the viral membrane as the key to initiating cellular viral infection. It is a homotrimeric glycoprotein that houses the receptor-binding domains (RBDs), promoting the first contact of the virus with the human ACE2 receptor via the receptor-binding motif (RBM).^4^^,^^5^ Cryo-electron microscopy (cryo-EM) has enabled the reconstruction of high-resolution structures of the spike in multiple states, bringing insights into how the virus gains access to the host cells.^6^^,^^7^ In the closed state, or RBD “3-down” conformation, the spike is shielded from the host immune system by the solvent-exposed glycans.^6^^,^^7^^,^^8^ In the “1-up” conformation, the RBM is exposed and binds specifically to the human ACE2 receptor.^4^^,^^5^ Thus, understanding the mechanism of the RBD opening, and how new mutations modulate the conformational ensemble of the RBD, is of interest to pharmaceuticals in terms of prevention and treatments.

Various experimental and computational studies have since provided insights into the spike’s structural dynamics. All-atom molecular dynamics (MD) simulations have elucidated essential features of the RBD opening, mainly of the wild-type (WT) variant.^9^ In the early days of the pandemic, D.E. Shaw Research provided microsecond-long unbiased all-atom simulations of the WT spike and other viral proteins, either alone or with protein partners or ligands.^10^ Community-powered Folding @ home simulations found the WT spike from SARS-CoV-2 to have a lower propensity to be in the 1-up conformation than SARS-CoV-1 and discovered cryptic druggable pockets in the extremely open state.^11^ Simultaneously, weighted ensemble MD simulations and the manifold embedding of cryo-EM particles showed that a single glycan site controls the gating of the RBD opening.^8^ Glycans have also been shown to modulate the population of the open state,^12^ mediate interdomain interactions,^13^ and provide a dynamic shield of epitope exposure.^14^ Free-energy surfaces of the WT opening, computed for the glycosylated and non-glycosylated spike, using replica-exchange umbrella sampling simulations, revealed that the glycan cover increases the free-energy barrier between the down/up conformations and stabilizes the inactive down state.^15^ In an attempt to explain the allosteric effect of mutations in the central and bottom parts of the spike, machine-learning analysis of MD trajectories showed that distant residues could modulate the RBD opening,^16^ while a structure-based coarse-grained and umbrella sampling simulational study elucidated the importance of interdomain RBD-NTD contacts along the opening pathway.^17^

Unfortunately, the virus evolves rapidly. Recent data show that emerging variants adapt their survival mechanisms to reinfect the immunized population and outwit current therapeutics.^18^^,^^19^^,^^20^ For example, the T372A mutation removes the glycosylation site N370 and thus promotes open RBD conformations.^21^ Image classification in cryo-EM experiments also showed that mutations can modulate the 3-down and 1-up populations.^22^^,^^23^^,^^24^ A notable single-molecule fluorescence resonance energy transfer (smFRET) experiment measured the timescale of the RBD opening of the WT spike and determined that it is on the order of seconds.^25^ Interestingly, the opening kinetics of the D614G mutant is significantly slower, raising the question whether the virus might optimize infectivity by modulating the kinetics of RBD opening rather than by evading immunity.^26^

Omicron BA.1 (or B.1.1.529) and its subvariants quickly superseded their predecessors around the world.^27^ Genomic and structural data suggest that the rapid antigenic evolution of Omicron helps the virus escape immune responses.^24^^,^^28^^,^^29^^,^^30^^,^^31^^,^^32^^,^^33^ Compared to WT, Omicron BA.1 exhibits 37 mutations, three deletions, and one insertion in the spike protein, and its sublineages may have a more extensive array of mutations. Some of these mutations stabilize the interactions between the S1 domain (containing the RBD) and the S2 domain, responsible for membrane fusion.^32^ As expected, many spike mutations, especially those concentrated in RBD, modulate the interactions with the ACE2 and antibodies.^33^ Recent studies show that Omicron RBD binds to the ACE2 receptor with similar or higher affinities compared to other variants, including the WT,^28^^,^^34^^,^^35^ suggesting that the genomic changes do not compromise receptor binding. Moreover, some mutations located at the interdomain RBD interfaces favor a more tightly packed conformation,^36^ potentially modulating the dynamics of RBD opening rather than the interactions with ACE2. Therefore, understanding the consequences of these mutations can elucidate Omicron’s evolutionary superiority. To address these critical issues, we performed detailed structural analyses for unbiased and biased all-atom MD simulations to probe the differences between WT and Omicron in conformations and dynamics during the RBD opening.

## Results

### Omicron mutations on the RBD-RBD interface

**Figure 1:**
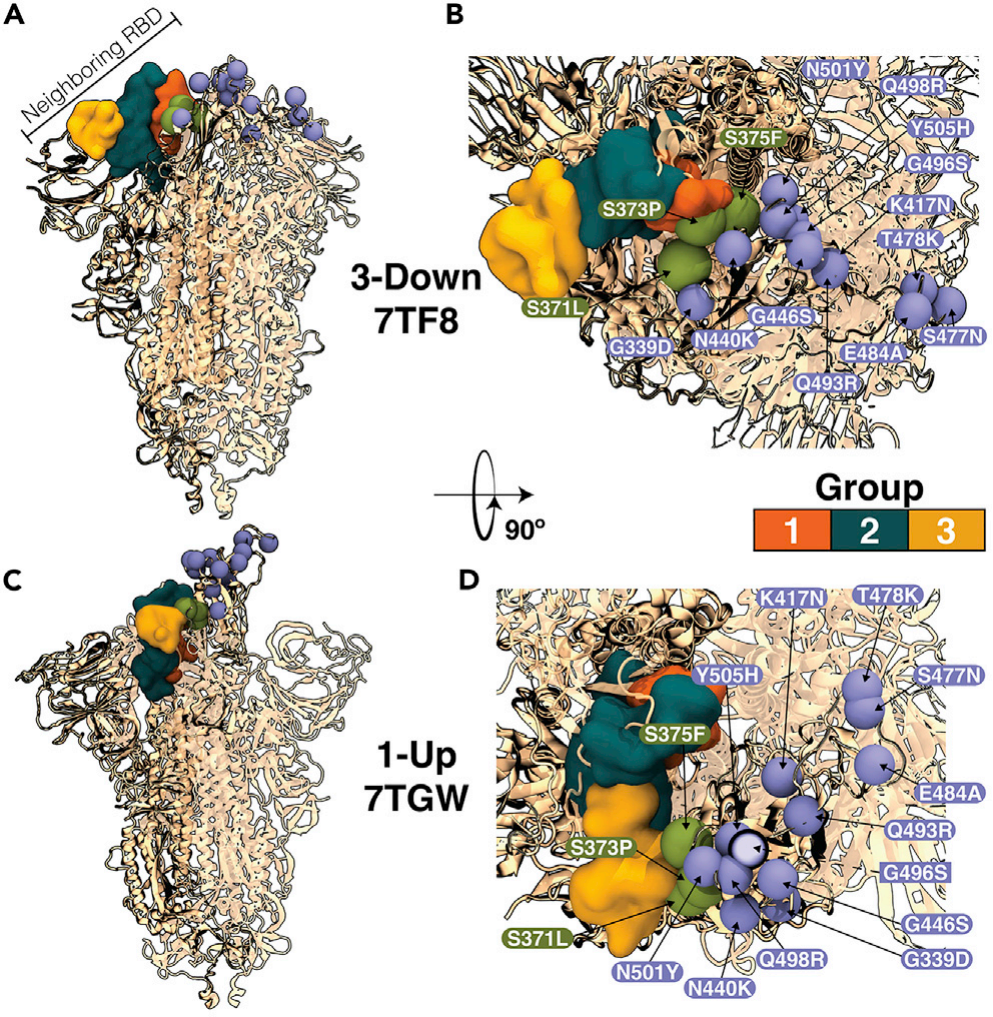
Omicron BA.1 in the 3-down and 1-up conformations (A and B). and B. PDB 7TF8^36^ shows Omicron in the 3-down conformation in rotated and zoomed top-view. (C. and D). show a 1-up conformation (PDB: 7TGW^23^) in similar perspectives. The RBD-specific mutations are presented using VDW presentation in VMD.^37^ Mutations K417N, N440K, G446S, S477N, T478K, E484A, Q493R, G496S, Q498R, N501Y, G339D, and Y505H are exposed to the solvent and shown as purple spheres. Omicron-specific mutations S371L, S373P, and S375F are buried in the 3-down state and shown as green spheres. We note that G339D is not present in 7TGW. The neighboring RBD interface is shown as a surface with the following color scheme: *Group 1* is in orange with residues 403 to 415 and 500 to 507; *Group 2* is in teal with residues 416 to 430, 446 to 462, and 491 to 498; and *Group 3* is in yellow with residues 470 to 490.

In Figure 1, we present the Omicron BA.1 spike extracted from cryo-EM structures in the 3-down (7TF8)^36^ and 1-up (7TGW)^23^ states. In both structures, Omicron partially exposes these mutations to the solvent: K417N, N440K, G446S, S477N, T478K, E484A, Q493R, G496S, Q498R, N501Y, G339D, and Y505H (shown as purple spheres). Meanwhile, Omicron RBD-specific mutations *S371L*, *S373P*, and *S375F* are completely buried in the 3-down conformation (shown as green spheres). For convenience, the mutations S371L, S373P, and S375F will be called the RBD three mutations. Interestingly, in the 1-up state, these RBD three mutations are contacting the neighboring RBM (the yellow group) that acts as a support for the RBD in the open state. To verify that the presence of these contacts is an endogenous pattern in the 1-up state, we analyzed multiple cryo-EM structures of the spike from various variants, as shown in Table S1, finding that in more than 90% of the solved structures, at least one residue of the triplet is making contact with the neighboring RBM. Because these mutations change from small and polar residues into bulkier and more hydrophobic ones located at the interdomain RBD interface, we hypothesized that they are involved in the conformational and dynamical changes of the RBDs along the opening. Indeed, Cerutti et al. superimposed the 3-down states of WT and Omicron, finding that Omicron is more tightly packed.^24^

### Free-energy landscape of RBD opening in WT and Omicron spike

To investigate the dynamical differences between WT and Omicron, we ran well-tempered multiple-walker metadynamics (mw-MetaD) simulations^38^^,^^39^ of the fully glycosylated spikes to explore the conformational landscape of RBD opening using Gromacs^40^ and PLUMED.^41^ We defined collective variables that guide the spike from the inactive (3-down) to the active (1-up) state. To parametrize this opening pathway, we collected a set of available trajectories of the WT spike that sampled the closed and open states and transitions between them.^8^^,^^10^^,^^13^ We found that the space spanned by two principal components (PC 1 and PC 2) captured well the opening transition as a rigid body movement. After a preliminary pathway optimization, we initialized 18 walkers from conformations equally spaced in the PC space for both variants (see STAR Methods and Figure S1).

**Figure 2:**
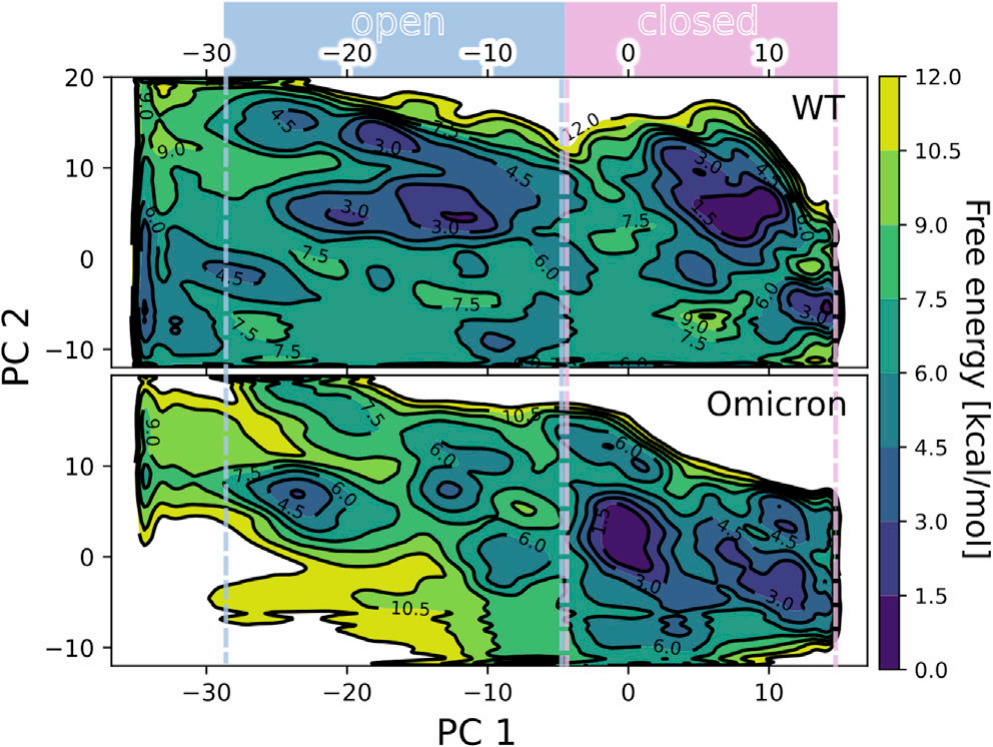
Free-energy surfaces along the opening pathway The 2D free-energy surfaces describe the energetics of the open-to-closed transition for the WT spike (top) and Omicron spike (bottom). Note shifts in the position of the main closed-state minima (deep blue basins on the right) and the depth of the open-state minima (blue spots in the central-to-left part of the plot), as well as the accessibility of the region corresponding to the bottom left corner of the graph.

The extracted free-energy landscape of the WT spike (top panel in Figure 2) features two major basins corresponding to the down (right) and 1-up (left) states, with their relative free energy only slightly (1–2 kcal/mol) in favor of the down state, and separated by a free-energy barrier of ca. 7 kcal/mol. These values are consistent with results from smFRET^25^ and umbrella sampling^15^ studies, giving us confidence in the reproducibility of our setup (Figure S2). On the other hand, the free-energy surface corresponding to the Omicron variant (bottom panel in Figure 2) features interesting differences compared to WT. The position of the main free-energy minimum in Omicron is at around PC 1 = 0 instead of at around 8 for WT with the closed substate of Omicron (PC 1 = 10) is still thermally accessible, having a 2 kcal/mol free-energy difference. The two open substates at PC 1 = −12 and PC 1 = −24 correspond to the similar substates for WT, but their overall stability is lowered by 2–3 kcal/mol, which is consistent with the more closed state of Omicron identified in cryo-EM experiments.^42^ The relative stability of these substates is also inverted, so that the dominant open state is more open (lower PC 1) in the case of Omicron. The opening pathway of Omicron is more heterogeneous and features more intermediates, but they are separated by barriers of similar height to those in the WT case, *i*.*e*., of 6–7 kcal/mol. Quite notably, the bottom left part of the plot (PC 1 lower than −10 and negative values of PC 2) is thermodynamically accessible in WT but not in Omicron, strongly suggesting that the Omicron mutations restrict the conformational heterogeneity of the RBD ensemble and make the open state better defined.

### Changes in RBD opening rates induced by omicron mutations

**Figure 3:**
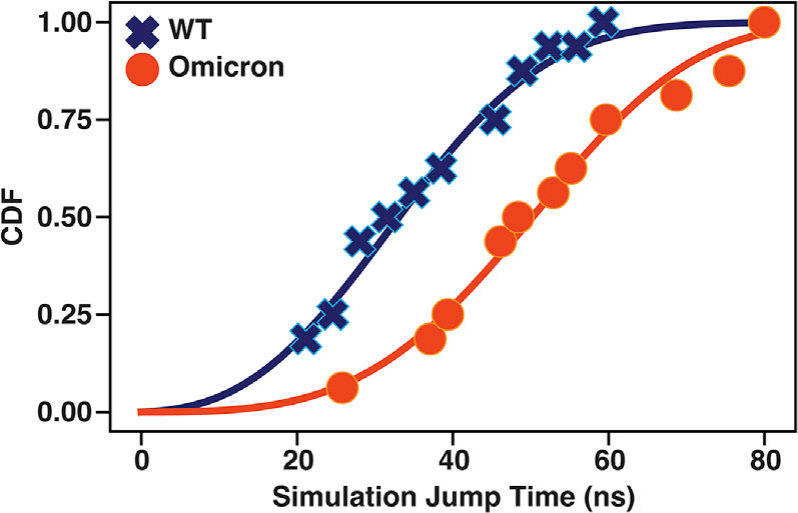
Extreme biasing MetaD simulations for WT and Omicron RBD opening Cumulative distribution function (CDF) as a function of time to reach the transition region (PC 1 = −10) for WT (navy blue crosses) and Omicron (red circles). Solid lines show the fit of the KTR theory,^43^ assuming a logarithmic time dependence of the biasing potential. Because these simulations are in the overbiasing regime, only qualitative order-rank comparison is possible.

As it is not trivial to extract even relative opening rates from free-energy profiles featuring multiple and often non-matching minima, we used the Kramers’ time-dependent rate (KTR) method for a qualitative comparison of the RBD opening times from biased simulations.^43^ Here, we ran 15 conventional MetaD (cMetaD) simulations starting from the down conformation (PC 1 = 10), biasing just PC 1 for both systems (for details see STAR Methods). Conventional MetaD was used to explore possible differences in the opening transition times under the same biasing conditions. These simulations were halted when they reached PC 1 = −10. In Figure S3, we plot PC 1 as a function of the simulation time for both systems. We find that WT diffuses more rapidly along PC 1; this is indicated by the slope of the PC 1 exploration as a function of time, with WT having a steeper slope. In Figure 3, we plot the cumulative distribution function (CDF) (*i*.*e*., the probability of reaching PC 1 = −10) as a function of the simulation jump time (see STAR Methods for details), showing that it is more probable for WT to reach the transition region more rapidly. We note that because these simulations were done under extreme biasing conditions, it is impossible to determine absolute values of the transition rates (see the overbiasing case in ref. ^43^). However, these results give us an estimate of the rank ordering of rates, assuming PC 1’s efficiency similar for both, Omicron has a smaller rate (see Figure S4). Interestingly, smFRET measurements also showed that for many variants (such as the D614G mutant, also present in Omicron), the RBD opens more slowly than in WT.^26^

### The effect of omicron mutations on contact patterns between neighboring RBDs

**Figure 4:**
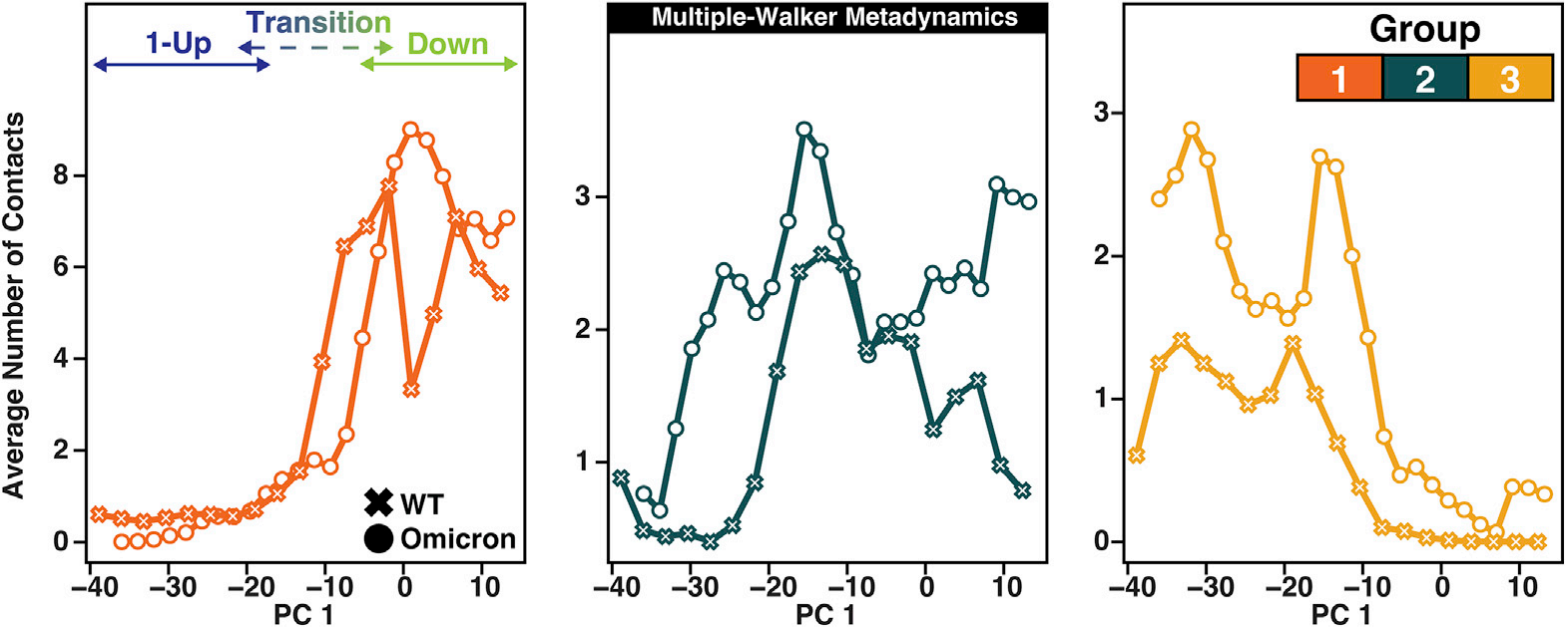
Average number of contacts formed by the three RBD residues **371, 373, and 375** with groups **1, 2, and 3** of the neighboring RBD (defined in Figure 1) as a function of PC 1 for the multiple-walker MetaD ensemble for WT (crosses) and Omicron (circles).

We now focus on the structural influences materialized by the RBD three mutations. The conformations from both the mw-MetaD and the cMetaD simulations can be treated as proxies of sample configurations along the RBD opening. To understand if the RBD three mutations are forming interdomain contacts along the opening process, we monitored the average number of contacts between the three mutations on the RBD and its neighboring RBD. We divided the neighboring RBD into three groups according to their spatial location in the closed state (Figure 1). For the mw-MetaD set, we calculated the average number of contacts (*i*.*e*., the number of contacts divided by the number of conformations in each bin; see the STAR Methods for details) formed by the RBD three mutations with the neighboring RBD along PC 1. The results for Omicron and WT are shown as circles and crosses, respectively, in Figure 4. For both systems, we found that these residues always form interdomain contacts with the neighboring RBD along the opening pathway (measured via PC 1), suggesting a sliding mechanism where the neighboring RBD acts as a support (or “handrail”) for RBD opening. The same observable was monitored for the cMetaD conformations (Figure S5), resulting in a behavior similar to that of the mw-MetaD. To confirm whether the sliding mechanism is an artifact due to overbiasing conditions or the quality of the chosen reaction coordinates, we also analyzed several publicly available WT opening trajectories. We found a similar mechanism (Figure S6). Impressively, those trajectories point to the same handrail behavior of the spike in both variants, with Omicron making many more interdomain contacts than WT on average. We also ran in-house unbiased MD simulations of both variants for 0.5 μ s starting from the down state. Although these unbiased simulations never reached the transition state, Omicron made twice as many contacts as WT per MD frame (Figure S7; see also Video S1). A possible reason why Omicron takes longer to reach the transition could be the sticky and bulky nature of the RBD three mutations. However, these are probably not the only Omicron mutations that change the flexibility of the opening of the RBD, as many distal mutations can also contribute to the cause allosterically.^16^

Video S1: Aligned conformations of the Omicron Spike from mw-MetaD simulations, showing a zoomed view of the RBD opening pathway The conformations were aligned according to PC 1 from closed to open. The three Omicron mutations (S371L, S373P, S375F) effectively make more contacts between two adjacent RBDs, specially at the interface with the 1-Up RBD. The movie was created with Molywood^54^ and VMD.^37^ related to figure 4

### Altered epitope exposure due to changes in RBD’s conformational ensemble in Omicron

**Figure 5:**
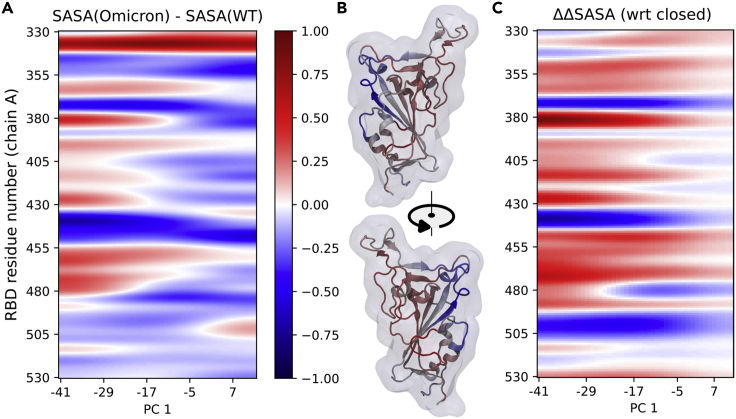
Changes in the exposure of RBD residues induced by the Omicron variant (A) Per-residue changes in solvent-accessible surface area (SASA) between the Omicron and WT spikes’ RBD (see STAR Methods for details). SASA for each residue is divided by the number of atoms in that residue. (B) Relative SASA changes offset by the mean in the closed state mapped on the RBD structure. (C) SASA changes offset by the values in the closed state, i.e., the mean value between PC1 = 0 and PC1 = 12. The mean value from PC1 = −30 to PC1 = −16 is color-coded on the structure in panel B.

These results show that Omicron is less flexible, more tightly packed, and stickier than WT, probably benefiting from stabilizing interdomain RBD contacts in both the 3-down and 1-up states. To study why this would be of evolutionary advantage, we calculated the differences in the solvent-accessible surface area of the RBD residues along the opening pathway conformations. As seen in Figure 5A, in the case of Omicron, certain regions (residues 368–375, 432–444, and 492–510) are much less exposed upon opening than they are in WT. When color-mapped onto the atomistic structure, these regions cluster on one side of the RBD (see Figure 5B), while the opposite side becomes correspondingly more solvent-exposed. Such a picture corresponds well with the above notion of a more restricted conformational ensemble of Omicron’s RBD and with the stabilizing effect of the RBD three mutations (371, 373, and 375). On a biological level, this difference in exposure can be linked to the less favorable binding of several antibody classes, one making frequent contacts with the 492–510 region and another contacting the 371–375 mutant triad (clusters 1 and 2, respectively, as defined by^44^). Also, consistently with the notion of reduced accessibility, several antibodies (DH1047, S2X259, and CR3022) targeting the affected region, virtually free of mutations, have their neutralization affinities lowered just slightly—by 8%–25%—as opposed to a more binary (on-off) result induced by altering the interface through mutations.^36^ Combined, these results strongly indicate that an altered exposure pattern can be used by evolution to limit the efficiency of acquired immunity.

## Discussion

In summary, we found significant differences between the conformational ensembles and dynamics of the spike’s RBD in WT and Omicron. While free-energy profiles showed a much more restricted conformational freedom of the Omicron RBD, our extreme biasing simulations showed that it also moves more slowly in the conformational space. These results are in qualitative agreement with smFRET experiments, where variants (such as the D614G) are slower to open. These suggest that newer variants might evolve to enhance the RBD kinetic stability and reduce its opening dynamics. Our cryo-EM structural analysis and MD simulations reveal a sliding mechanism for RBD opening where the neighboring RBD acts as handrail support. Moreover, substituting polar for hydrophobic residues at positions 371, 373, and 375 increases Omicron’s interdomain contacts along the opening pathway.

Interestingly, for all new Omicron variants (BA.2 to BA.5), residues 373 and 375 remain the same, while S371 mutates to phenylalanine, an even bulkier and more hydrophobic residue (Figure S8). Experimental studies of the Omicron BA.2 variant show that these residues create a tighter packing and more interdomain contacts in the down conformation compared to BA.1,^45^ where the RBD three mutations might favor the tight packing. Ultimately, we find that Omicron’s enhanced compactness and stickiness lead to a conformational ensemble that affects epitope exposure through altered solvent accessibility, suggesting a possible mechanistic link between the mutation-modulated RBD conformational ensemble and immune evasion.

### Limitations of the study

Due to the setup of our free-energy simulations, involving physical restraints on the S2 part of the spike protein, we were unable to observe major allosteric effects mediated by the S2 part, effectively limiting our analysis of domain dynamics to direct interactions between the S1 domains. Any external chemical modifications that could differentially affect the spike, such as hydrolysis or post-translational modifications (other than the standard glycan shield), were also not considered. Moreover, all limitations of classical molecular dynamics apply, such as fixed protonation states or chemical topology, finite simulation time, and approximations inherent in modeling molecular interactions in the force field. For future work, it would be intersting to analyze the ranking of the conformational ensembles with stability-prediction tools^46^, and study the effects of using different collective variables for the enhanced sampling^43^.

## STAR★Methods

### Key resources table

**Table:** 

REAGENT or RESOURCE	SOURCE	IDENTIFIER
**Deposited data**		

Setup for the metadynamics simulations	This paper	github.com/milafternoon/plumed-spike-omicron
Plumed inputs	This paper	plumed-nest.org/eggs/22/040/

**Software and algorithms**		

Gromacs 2021.4	^1^Abraham et al. 2015^40^	gromacs.org/Downloads
Plumed 2.8.0	^2^Tribello et al. 2014^41^	plumed.org/doc-v2.8/user-doc/html/index.html
Python 3.9	Python Software Foundation	anaconda.org

### Resource availability

#### Lead contact

Further information and requests for resources and reagents should be directed to and will be fulfilled by the lead contact, Pilar Cossio (pcossio@flatironinstitute.org).

#### Materials availability

This study did not generate new unique reagents.

#### Data and code availability

The setup of the metadynamics runs is available at github.com/milafternoon/plumed-spike-omicron, and metadynamics inputs are available on PLUMED-NEST at plumed-nest.org/eggs/22/040. This paper does not report original code. Any additional information required to reanalyze the data reported in this paper is available from the lead contact upon request.

### Method details

#### Molecular dynamics setup

The glycosylated Spike WT protein was taken from the CHARMM repository^47^ (parametrized with CHARMM36m^48^ and TIP3P water). Spike Omicron point mutations were introduced in the conformations and topology file using Gromologist.^49^ Omicron deletions and insertions at positions 69–70 and 212/215 were ignored as their location implied they are solely involved in escape from NTD-targeting antibodies. The full system, contained in a rectangular 19.22×19.22×19.95 nm box, featured 226,799 water molecules, 682 potassium and 694 chloride ions. Positions of the α-carbon atoms of the S2 part (residues 700–1146) were restrained throughout the simulations in order to use an absolute geometric reference for the principal component analysis (PCA) calculation. A standard NPT protocol involving the CSVR (V-rescale) thermostat^50^ with a bath temperature of 300 K and the new C-rescale barostat^51^ set to 1 bar was used. Bonds involving hydrogen atoms were restrained to use the standard 2-fs timestep. Electrostatics was treated with PME with a 1.2 nm cut-off for the direct-space part.

#### PCA coordinates and initial configurations

To define the principal component (PC) space, we used (a) 3 in-house targeted MD simulations of RBD closing, (b) the 1-up trajectory from RIKEN,^13^ (c) the WE trajectory from Rommie Amaro’s group^8^ and (d) 6 trajectories published by DE Shaw Research.^10^ For each trajectory, the S2 part was aligned three times with permuted chains, so that both the open and closed RBDs were used for PCA (thanks to the permuted alignments, the RBD was always in the same position with respect to the central S2 core). Spline interpolation was used to generate reference coordinates for the initial path.

We then used the string method^52^^,^^53^ to define a pre-optimized path with initial configuration of the opening transition. 150 trajectories with 50-ps long iterations were used with 44 points along the string and 12 runs per swarm. The positions on the 2D PCA plane were used to define the path in PLUMED.^41^ These relaxed and optimized intermediates, mapping the conformational transition in fine detail, allowed us to calculate representative conformations of the opening, and further use PC1 and PC2 as collective variables for the multiple-walker well-tempered MetaD.

#### Multiple-walker well-tempered MetaD

For each system, we ran 18 walkers for 600 ns each, using Gromacs^40^ coupled with PLUMED,^41^ and the PCA projection (the PCAVARS variable defined by two pairs of reference structures) as the collective variable. The initial Gaussian had a width of (1.0, 0.5) PC unit and a height of 0.2 kJ/mol, with a deposition rate of 1 ps ${}^{-1}$. A biasfactor of 15 was used. The PCA projection was calculated for the single RBD domain alone, with the S2 part of the Spike trimer constrained using the position restraint option of Gromacs as noted above. In this way, the SIMPLE protocol in PCAVARS could be used instead of the OPTIMAL alignment-based calculation.

#### Conventional MetaD starting from the down conformation

We ran 15 conventional MetaD simulations starting from the down conformation with the same MetaD parameters for WT and Omicron, guaranteeing the same biasing conditions for both. As the single CV, we used PC1 as defined above. The Gaussian height and width were 0.03 kJ/mol and 2 PC units, respectively. The bias deposition time was 2ps. Gromacs 2021 with the PLUMED patch were used with the same MD parameters as described above. The simulations were halted the first time they reached PC1 = −10.

### Quantification and statistical analysis

#### Multiple-walker well-tempered MetaD

Due to the use of an asynchronous simulation protocol, free-energy convergence in the top panel of Figure S2 was calculated by subtracting a specified number of Gaussian kernels from the bottom of each HILLS file. The free-energy error in the central panel of Figure S2 was calculated using the block analysis method, as described in PLUMED documentation.

#### Conventional MetaD starting from the down conformation

The empirical CDF is calculated using the simulation time of each run to reach this value for the first time (for details see STAR Methods of ref.^43^). Assuming a logarithmic function of the time-dependence of the biasing potential, we use the analytical expression of the survival probability (Supplementary Equation 12 from ref.^43^) to provide a visual guide of the fit to the empirical CDF. We note that because we are in the overbiasing regime it is not possible to extract exact values of $k_{0}$ or of the efficiency of CV γ, but we can use Supplementary Equation 16 from ref.^43^ to compare $k_{0}^{*}\left(\gamma \right)$ for both systems.

#### Contact-structural analysis

To compare the average number of contacts formed at the WT and Omicron RDB-interface along the opening PC1 pathway for converged mw-MetaD, unconverged overbiased MetaD trajectories, and unconverged unbiased opening trajectories, we created bins along the PC1 axis, and assigned structures to the individual bins. We estimated the average number of contacts at each PC1-bin dividing the total number of contacts (for the frames in the bin) by the total number of frames in the bin, assuming that there are no dramatic free-energy changes along PC2 for each PC1 bin. This enables a qualitative comparison of the behavior of this observable between converged and unconverged opening trajectories. To validate this assumption, in Figure S9, we show the average number of contacts for the mw-MetaD WT and Omicron calculated by reweighting the observable along PC2 using the converged free-energies (Figure 2). We find similar results to those without reweighting with Omicron forming more contacts on average than WT for all groups (shown in Figure 1).

#### Solvent-accessible surface area calculation

The per-residue SASA was calculated for conformations generated in the mw-MetaD scheme using an automated workflow implemented in the BioExcel COVID-19 webserver (bioexcel-cv19-dev.bsc.es) for WT and Omicron. In brief, ca. 200 equally time-spaced frames were selected from each walker’s trajectory, and the gmx sasa tool is used to calculate per-atom SASA. These values were combined to calculate a residue-wise averaged per-atom surface area (i.e., summing surface areas from individual atoms that comprise each residue, and dividing by the total number of atoms for each residue). Subsequently, average values along PC1 bins are obtained by weighted averaging, using the free-energy surface shown in Figure 2 as (unnormalized) weights (similarly as for Figure S9).
