# Supplementary material for: Omicron mutations increase interdomain interactions and reduce epitope exposure in the SARS-CoV-2 spike
Source: iScience. 2023 Jan 20;26(2):105981. doi: 10.1016/j.isci.2023.105981 (PMC9851991; doi:10.1016/j.isci.2023.105981)
Supplement: Document S1. Figures S1–S9 and Table S1 [file mmc1.pdf]

iScience, Volume *26*

Supplemental information

Omicron mutations increase interdomain interactions and reduce epitope exposure in the SARS-CoV-2 spike

Miłosz Wieczór, Phu K. Tang, Modesto Orozco, and Pilar Cossio

Supplementary Figures

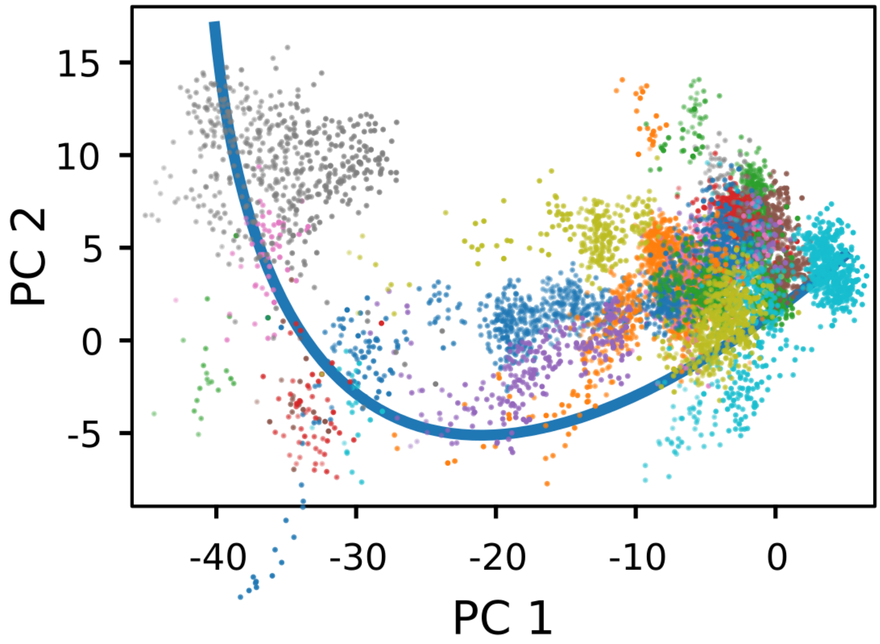

Supplementary Figure 1: **PCA projection of the initial ensemble of trajectories used to define the seeding structures for string-based pathway optimization, related to Figure 2.** Included are 1-up trajectories from RIKEN[1] and DE Shaw Research,[2] as well as transition pathways sampled by us (4 200-ns steered MD simulations) and Amaro's group (Weighted Ensemble simulations featuring the extreme opening in blue).[3] The blue line represents the initial trajectory in the PC space resulting from parametric spline interpolation of the data points.

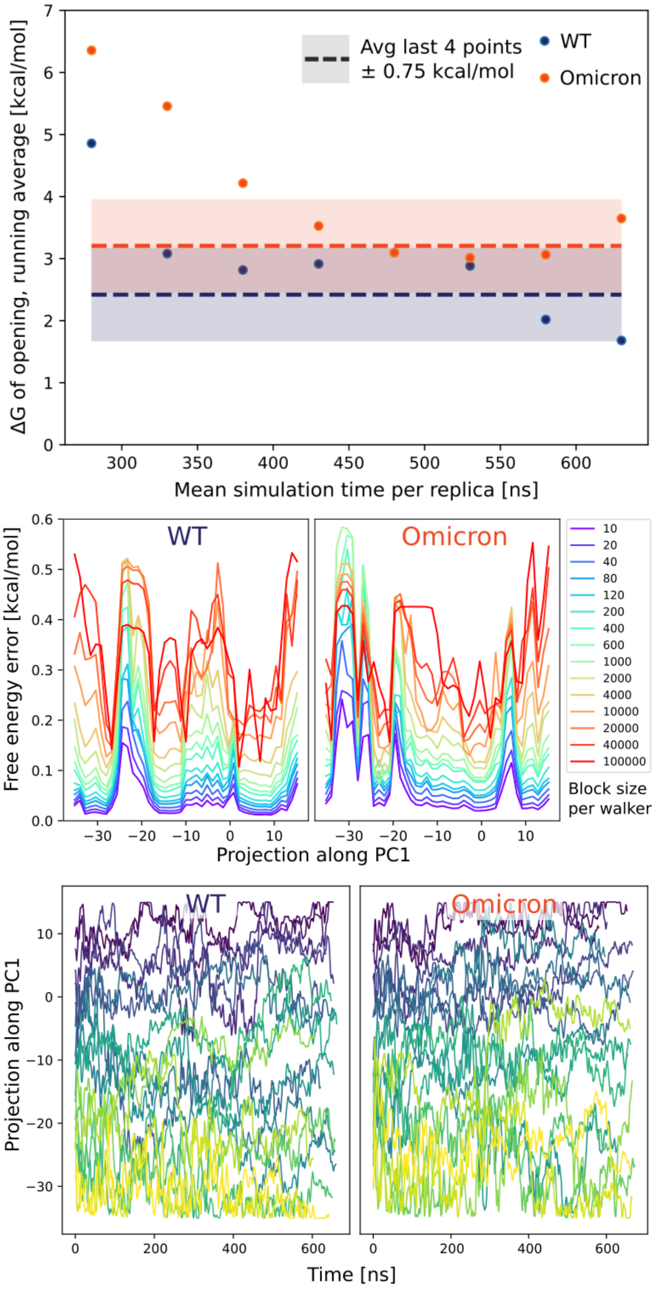

Supplementary Figure 2: **Assessing the convergence of the free energy calculations, related to Figure 2.**Top: Convergence of the multiple walker well-tempered metadynamics. Shown are running average values of the opening free energy, averaged over the previous 150 ns per-replica (e.g. the value for 630 ns corresponds to an average from 480 to 630 ns per replica). The free energy profile in Fig. 2 corresponds to the rightmost data point. Middle: Error in the free energy estimate along PC1 calculated from block averaging. The calculation was performed using dynamically calculated weights, as introduced in [4]. Prior to block averaging, data from individual walkers was combined and sorted by simulation time. Bottom: Time evolution of the PC1 projection for individual walkers (a total of 18 per system), Gaussian-smoothed to enhance visibility of individual trajectories.

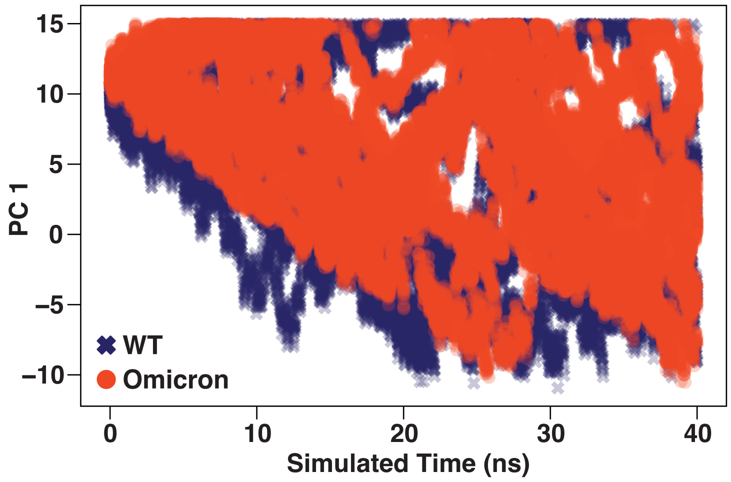

Supplementary Figure 3: **Extreme biasing MetaD simulations for WT and Omicron RBD opening, related to Figure 3**. PC 1 as a function of time for 15 conventional MetaD simulations starting from the 3-down conformation (PC 1=10) and biasing PC 1 for WT (crosses) and Omicron (circles).

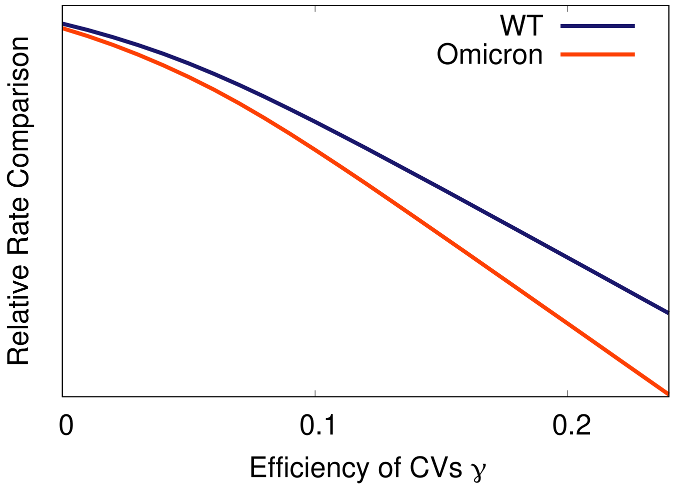

Supplementary Figure 4: **Relative rates of RBD opening between WT and Omicron, related to Figure 3.** WT (blue) and Omicron (orange) relative transition rate comparison as a function of the efficiency of CVs parameter γ given by Eq. S16 from the Supplementary Information of ref.[5] Because these simulations are performed under extreme biasing conditions, it is not possible to determine the exact value of the rate or γ but only compare $k_0^*(\gamma)$.

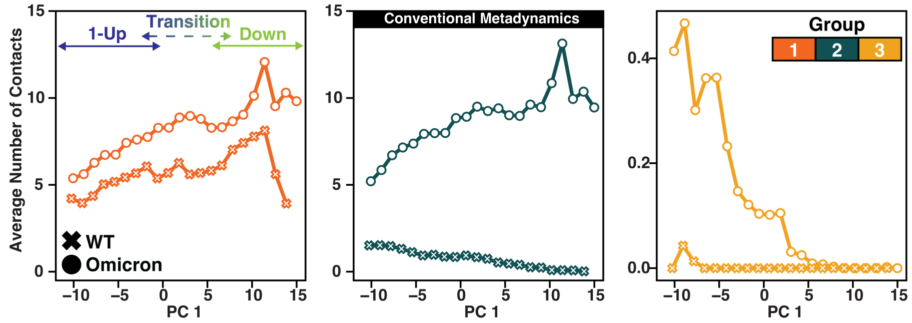

Supplementary Figure 5: **Average number of contacts profile from conventional metadynamics, related to Figure 4**. Average number of contacts formed by 371, 373 and 375 with groups 1, 2 and 3 (defined in Figure 1 Main Text) for the overbiasing MetaD opening simulations as a function of PC1 for WT (crosses) and Omicron (circles).

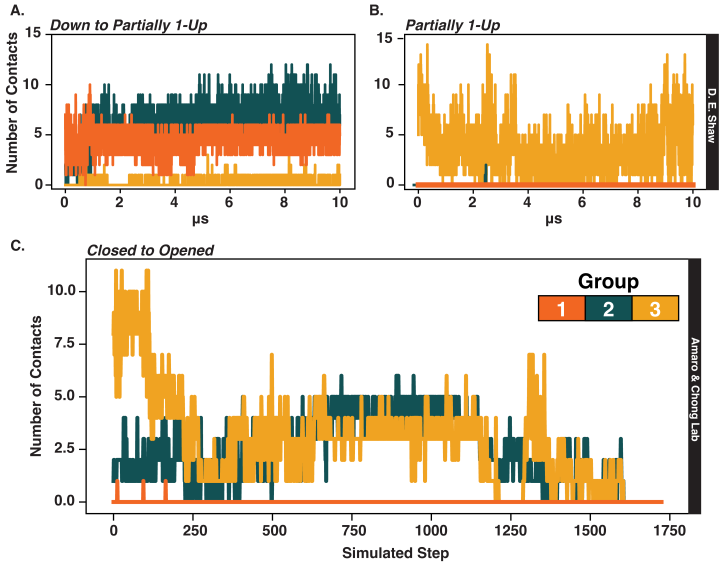

Supplementary Figure 6: **Average number of contacts profile from in-house and public data, related to Figure 4**. Average number of contacts over time formed by 371, 373 and 375 with groups 1, 2 and 3 (defined in Figure 1 Main Text) for the WT Spike from **A.** in-house unbiased simulations **B.** Amaro and Chong Lab[3] **C.** D.E. Shaw[2] .

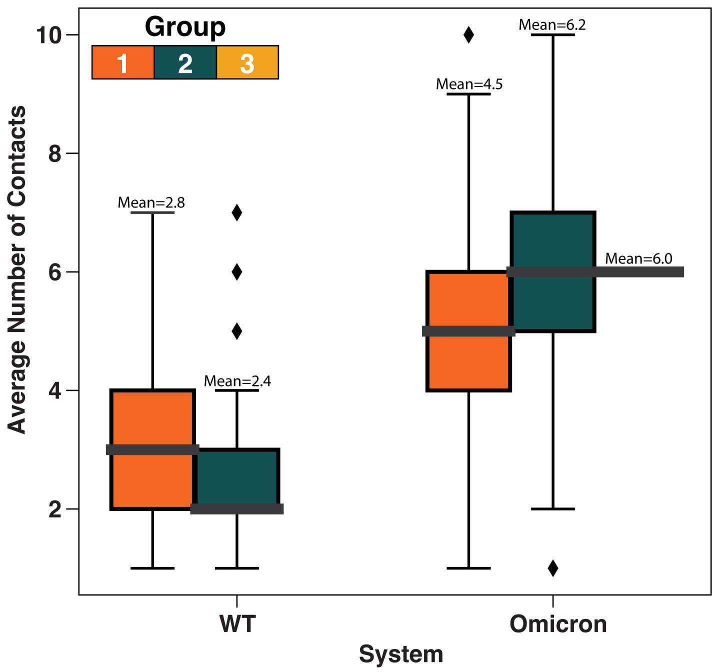

Supplementary Figure 7: **Average number of contacts profile for unbiased MD simulations, related to Figure 4.** Average number of contacts formed by 371, 373 and 375 with groups 1, 2 and 3 (defined in Figure 1 Main Text) for unbiased MD simulations starting in the down state for WT and Omicron, which ran for 500 ns. The grey lines are medians. The diamonds are outliners.

	358 378
**Wuhan-Hu-1**	**ISNCVADYSVLYNSASFSTFK**
**BA.1 I NCBI I UFO69279.1**	**ISNCVADYSVLYNLAPFFTFK**
**BA.2 I 7XIX**	**ISNCVADYSVLYNFAPFFAFK**
**BA.3 I 7XIY**	**ISNCVADYSVLYNFAPFFTFK**
**BA.4 I 7XNQ**	**ISNCVADYSVLYNFAPFFAFK**
**BA.5 I NCBI I UOZ45804.1**	**ISNCVADYSVLYNFAPFFAFK**

Supplementary Figure 8: **Mutation trend among COVID-19 variants, related to Figure 5.** Sequence alignment of SARS-CoV-2 Omicron subvariants from BA.1 to BA.5 from residues 358 to 378 against the Wuhan-Hu-1 (WT). WT residues S371, S373, S375 are highlighted in green. These residues are mutated to bulkier and more hydrophobic ones (red highlighted).

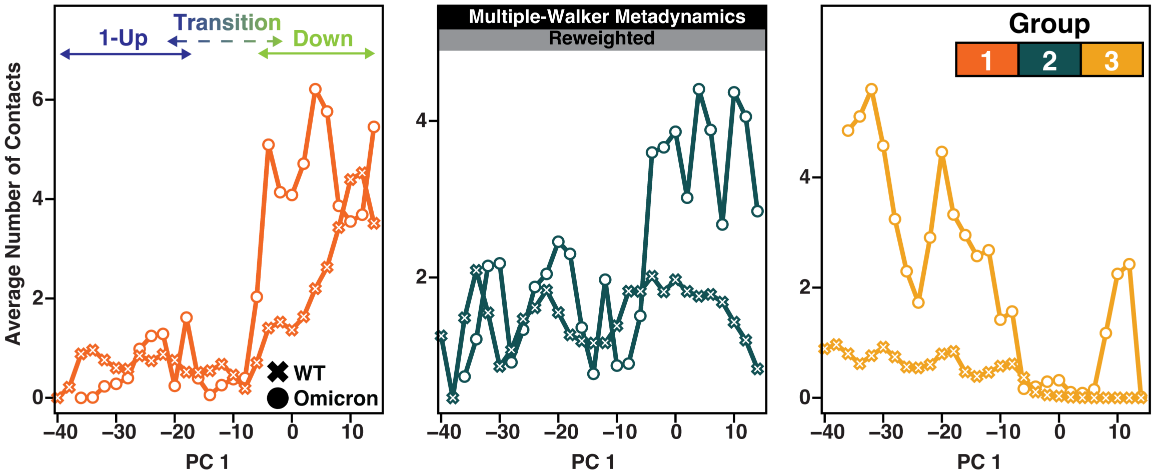

Supplementary Figure 9: **Group-wise decomposition of average contact numbers in multiple-walker metadynamics simulations, related to Figure 4.** The plot is analogous to Figure 4, but here the within-bin averaging has been performed according to weights dependent on the free energy along PC2 (see Methods).

Supplementary Tables

VARIANT	PDB ID	REFERENCE	Neighboring RBM in contact with 371 373 375 (heavy atom <10 A)
WT	6VSB	Wrapp, D., et al (2020) Science 367: 1260-1263	Neighboring RBM not resolved
	6XM3	Zhou, T., et al (2020) Cell Host Microbe 28: 867-879.e5	Yes
	6XM0	Zhou, T., et al (2020) Cell Host Microbe 28: 867-879.e5	Yes
	6XM4	Zhou, T., et al (2020) Cell Host Microbe 28: 867-879.e5	Yes
	6Z97	Huo, J et al. (2020) Cell Host Microbe 28: 445-454.e6	Yes
	7DDN	Zhang C (2021) Nat Commun 12: 264-264	No
	7AD1	Juraszek J 2021) Nat Commun 12: 244-244	No
	7DZW	Liu Y (2021) Cell 184: 3452-3466.e18	Yes
	6ZGG	Wrobel (2020) Nat Struct Mol Biol 27: 763-767	Yes
OMICRON	7WK9	Hong, Q., Han, W., Li, J. et al. Nature 604, 546–552 (2022).	Yes
	7WK3	To be published.	Yes
	7TGW	Ye, G et al (2022) Nat Commun 13: 1214-1214	Yes
	7WVN	Hong, Q., Han, W., Li, J. et al. Nature 604, 546–552 (2022).	Yes
	7WVO	Hong, Q., Han, W., Li, J. et al. Nature 604, 546–552 (2022).	Yes
	7TGX	Ye, G et al (2022) Nat Commun 13: 1214-1214	Yes
	7TL9	Gobeil, S.M., et al. (2022) Biorxiv DOI:	Yes
	7TEI	Gobeil, S.M., et al. (2022) Biorxiv DOI:	Yes
	7TB4	Wang, L.,et al (2021) Biorxiv DOI: 10.1101/2021.02.25.432969	Yes
Mink	7R1B	Wrobel, A.G. et al. (2022) Nat Commun 13: 1178-1178	Yes
D614G	7EB3	Yang et al. (2021) J Biol Chem 297: 101238-101238	Yes
	7EB0	Yang et al. (2021) J Biol Chem 297: 101238-101238	Yes
	7KEA	Gobeil. (2021) Cell Rep 34: 108630-108630	Neighboring RBM not resolved
	7KE9	Gobeil. (2021) Cell Rep 34: 108630-108630	Neighboring RBM not resolved
	7KDL	Gobeil. (2021) Cell Rep 34: 108630-108630	Neighboring RBM not resolved
	7KDJ	Gobeil. (2021) Cell Rep 34: 108630-108630	Neighboring RBM not resolved
	7KDH	Gobeil. (2021) Cell Rep 34: 108630-108630	Neighboring RBM not resolved
Kappa	7VXI	Wang Y (2021) Nat Commun 12: 7345-7345	Yes
	7VXE	Wang Y (2021) Nat Commun 12: 7345-7345	Yes

Supplementary Table 1: **Structural analysis of Spike PDBs in the 1-up conformation, related to Figure 1** Table showing the PDB ID, Variant, reference, and whether residues 371, 373, 375 are in contact with the neighboring RBM. A contact is made if any pair of heavy atoms come within less than 10 Å of each other. Green rows correspond to structures where the interaction is formed, red rows – not formed, and grey rows indicate structures in which the RBM has not been resolved experimentally.

References

(1) Mori, T., Jung, J., Kobayashi, C., Dokainish, H. M., Re, S., and Sugita, Y. (2021). Elucidation of interactions regulating conformational stability and dynamics of SARS-CoV-2 S-protein. Biophysical journal, 120, 1060–1071.

(2) D. E. Shaw Research (2020). Molecular Dynamics Simulations Related to SARS-CoV-2. D. E. Shaw Research Technical Data.

(3) Sztain, T., Ahn, S.-H., Bogetti, A. T., Casalino, L., Goldsmith, J. A., Seitz, E., McCool, R. S., Kearns, F. L., Acosta-Reyes, F., Maji, S., et al. (2021). A glycan gate controls opening of the SARS-CoV-2 spike protein. Nature Chemistry, 13, 963–968.

(4) Tiwary, P. and Parrinello, M. (2015). A time-independent free energy estimator for metadynamics. Journal of Physical Chemistry B, 119, 736–742. ISSN 15205207.

(5) Palacio-Rodriguez, K., Vroylandt, H., Stelzl, L. S., Pietrucci, F., Hummer, G., and Cossio, P. (2022). Transition Rates and Efficiency of Collective Variables from Time-Dependent Biased Simulations. The Journal of Physical Chemistry Letters, 13, 7490–7496.
